# Consequences of adolescent drug use

**DOI:** 10.1038/s41398-023-02590-4

**Published:** 2023-10-06

**Authors:** Michael R. Steinfeld, Mary M. Torregrossa

**Affiliations:** 1 Department of Psychiatry, University of Pittsburgh, 450 Technology Drive, Pittsburgh, PA 15219 USA; 2 Center for Neuroscience, University of Pittsburgh, 4200 Fifth Ave, Pittsburgh, PA 15213 USA

**Keywords:** Addiction, Scientific community

## Abstract

Substance use in adolescence is a known risk factor for the development of neuropsychiatric and substance use disorders in adulthood. This is in part due to the fact that critical aspects of brain development occur during adolescence, which can be altered by drug use. Despite concerted efforts to educate youth about the potential negative consequences of substance use, initiation remains common amongst adolescents world-wide. Additionally, though there has been substantial research on the topic, many questions remain about the predictors and the consequences of adolescent drug use. In the following review, we will highlight some of the most recent literature on the neurobiological and behavioral effects of adolescent drug use in rodents, non-human primates, and humans, with a specific focus on alcohol, cannabis, nicotine, and the interactions between these substances. Overall, consumption of these substances during adolescence can produce long-lasting changes across a variety of structures and networks which can have enduring effects on behavior, emotion, and cognition.

Adolescence is a period of critical development in the brain and body. Developmental changes in the brain lead to adolescents exhibiting heightened impulsiveness, which can lead to risky behaviors that may have long-term consequences [1, 2]. In particular, the use of both licit and illicit substances in adolescence can produce both acute and enduring effects on brain function and behavior. Of great concern is the fact that the prevalence of substance use disorders as an adult is greater if substance use is initiated during adolescence [3], however, other issues can persist into adulthood both related and unrelated to continued use. Alcohol, cannabis, and nicotine are among the most commonly used substances in adolescents, in part due to their availability, perceived lack of risk, and use in social settings [4].

Importantly, these substances act on receptors widely expressed in the brain (i.e. dopamine, GABAergic, and glutamate receptors), particularly in regions important for reward and cognition. Moreover, these receptor systems and brain regions undergo critical developmental changes during adolescence including a reduction in gray matter volume (GMV) [5] accompanied by an increase in white matter volume [6], changes in connections from subcortical to cortical circuits for emotional control [7], elimination of excess neural connections [8], refinement of the GABAergic system in the neocortex [9, 10], increases in dopamine (DA) receptor expression [11, 12], and development of the mesocorticolimbic system [13], to name a few. The molecular and structural changes in the brain are accompanied by changes in mood, behavior, and cognition, including heightened reward sensitivity [14], reduced inhibitory control [15, 16], and deficits in executive function relative to adults [17]. Furthermore, increases in sex hormones, such as testosterone and estrogen, have been shown to influence the brain’s response to reward [18, 19]. These changes in the brain and behavior make adolescents particularly likely to engage in substance use and susceptible to the long-term negative consequences of drug use. Given the potential societal impact of adolescent drug use, a number of researchers have investigated the long-term consequences of adolescent drug exposure in both clinical and preclinical studies. Indeed, several recent reviews have highlighted much of this research [20–25], so the present review is focused on the most recent work investigating the consequences of adolescent use of alcohol [Tables 1 & 5], cannabis [Tables 2 & 5], nicotine [Tables 3 & 5], or polysubstance combinations [Tables 4 & 5] in the human, non-human primate, and rodent literatures.

**Table 1:** Results and details of recent animal studies investigating AAE.

Reference	Species	Sex	Dose, Route of Administration	Age of Exposure (PND)	Age at Test(s) (PND)	Behavior Category	Behavior Test(s)	Behavioral Results	Brain Region(s)	Results
Maldonado-Devincci & Kirstein [32]	Rat	M, F	.75, 1.5 g/kg, i.p	30–50	65–80	Drug Taking	Oral s-a	↑ alcohol s-a in adolescent female rats given 1.5 g/kg alcohol exposure		
Maldonado-Devincci et al. [33]	Mouse	M, F	Vapor exposures, 10 liters/min	28–42	49–81, 70–102	-Drug Taking	-2 BC -OF	-↑ alcohol s-a in males after short abstinence -↓ alcohol s-a in females after long abstinence		
Lee et al. [40]	Mouse	M	Oral s-a, 5–40%	28–41	70	Depression	FST, sucrose preference test	↑ DLB	NAc shell, CeA	↑ mGlu1express in shell
Lee et al. [41]	Mouse	M	Oral s-a, 5–40%	28–41	70	-Anxiety -Depression	-LDB -FST	↑ ALB and DLB		
Sampedro-Piquero et al. [42]	Mouse	M	Oral s-a, 15%	21–52	58, 78	Anxiety	EZM	↑ ALB	PFC, Hippocampus, CeA, hypothalamus, BLA	-↓ BDNF Expression -↑ GR density -↑ CRF density
Van Hees et al. [43]	Mouse	M, F	Oral s-a, 20%	29–40	43, 80	-Anxiety -Depression -Drug Taking	-OF, EPM -FST -2 BC	-↑ Increased ALB, DLB, and alcohol consumption at adulthood	PFC	No change in microglia activation
Brancato et al. [42]	Rat		Oral s-a, 25%	35–54	64	-Anxiety -Depression	-Social Interaction Task -FST	-↑ ALB -↑ DLB	PVN, NaC	-↑ CRF expression -↑ D1 expression -↓ D2 expression
Pandey et al. [50]	Rat	M	2 g/kg, 20% i.p.	28–41	92	-Anxiety -Drug Taking	-EPM -Alcohol Consumption	-↑ ALB -↑ alcohol consumption	Amygdala	-↑ HDAC activity -↓ BDNF and Arc expression
Bohnsack et al. [52]	Rat	M	2 g/kg, 20% i.p.	28–41	120	-Anxiety -Drug Taking	-EPM, LDB -2 BC	-Impaired performance -↑ alcohol consumption	CeA	↓ Arc mRNA expression
Lawson et al. [54]	Mouse	M, F	Oral s-a, 20%	39.5–44.5	68.5	Memory	Conditioned freezing	↓ extinction recall	PL	↓ dendritic complexity
Kyzar et al. [51]	Rat	M	2 g/kg	28–41	100	Anxiety	EPM, LDB	↑ ALB	CeA	↓ Arc enhancer ↓ Arc mRNA expression RNA expression
Salling et al. [55]	Mouse	M	Oral s-a, 15%	30–60	64–80	Working memory	Nonmatch to Sample	Impaired performance	PL	-Hyperpolarization of resting membrane potential
Fernandes et al. [56]	Rat	F	Intragastric administration 3 g/kg, 20%	35, 35–58, 35–86	35, 49, 58, 72, 86, 100	-Anxiety -Working memory	-OF, EPM -NOR	↑ ALB Impaired performance	Hippocampus	-↓ BDNF expression -↑ GFAP level
Jury et al. [58]	Mouse	M	Vapor Inhalation + IP injection 19–22 mg EtOH/I + 1.5 g/kg, 20%	35–63	64				IL, BLA	-↓ IL neuronal spine density -↑ head width of BLA spines
Galaj et al. [59]	Rat	M	Intragastric administration 4 g/kg	28–45	47, 66				PL	-Modified L5 PNs -↑ intrinsic excitability of L5 PNs
Silva-Gotay et al. [60]	Rat	M, F	Oral s-a 10%	28–42	43				mPFC	-↑ gene expression of pro-inflammatory factors -↑ in TLR4 gene expression in males
Broadwater et al. [61]	Rat	M	Intragastric administration 5 g/kg, 25%	25–54	78.5				Frontolimibic regions	↓ resting-state connectivity between PFC and subregions and PFC-striatal regions
Tavares et al. [62]	Rat	M, F	Oral s-a, 10%	28–42	43				CG1	-↓ myelinated fiber density in males -No effect in females
Vetreno & Crews [64]	Rat	M	Intragastric administration 5 g/kg, 20%	25–55	56, 80, 220	-Working memory -Anxiety	-NOR -OF	-Impaired performance -↑ ALB	DG	-↓ neurogenesis -↑ cell death
Mulholland et al. [66]	Rat	M	Intragastric administration 5 g/kg, 35%	30–46	70				Hippocampus	-↓ dendritic spine density -Altered dendritic spine morphology
Healey et al. [68]	Rat	M, F	Intragastric administration 5 g/kg, 35%	30–46	70				CA1	-↓ dendritic spine density -Altered dendritic spine morphology
Risher et al. [70]	Rat	M	Intragastric administration 5 g/kg, 35%	30–46	47				Hippocampus	↑ astrocyte reactivity
Nwachukwu et al. [71]	Rat	M, F	Intragastric administration 5 g/kg, 35%	31–46	77				Hippocampus	-↑ astrocyte activation -↑ in GFAP+ cells
Healey et al. [72]	Rat	M	Intragastric administration 5 g/kg, 35%	31–46	74				Hippocampus	↓ astrocytic-synaptic contact
Swartzwelder et al. [73]	Rat	M	Intragastric administration 5 g/kg, 35%	30–46	70–75				Hippocampus	Alterations in GluN2B proteome
Swartzwelder et al. [74]	Rat	M	Intragastric administration 5 g/kg, 35%	30–46	71				CA1	↑ NMDA receptor-mediated evoked EPSCs
Walker et al. [77]	Rat	M	Intragastric administration 5 g/kg, 35%	30–45	46, 72				VO-PFC	-↑ intermediate dendritic spines -↓ in mature dendritic spines
Peng & Nixon [80]	Rat	M	Intragastric administration 5 g/kg, 35%	34–38	38, 40, 45, 52				Hippocampus, entorhinal cortex	↑ microglia activation
Hu et al. [81]	Mouse	M	Intragastric administration 3.5 g/kg, 25%	28–42	63	Depression	FST, Sucrose preference test	↑ DLB	DG	Apoptosis and dystrophy of microglia
Marshall et al. [82]	Rat	M	Intragastric administration 5 g/kg, 25% (titrated)	35–39	39				DG	-↑ microglial dystrophy -↓ BDNF expression
Agoglia et al. [87]	Mouse	M	Oral s-a, 20%	28–42	36–42				CB1 receptors	↓ alcohol s-a following inhibition of CB1 receptors
Sánchez-Marín et al. [89]	Rat	M	Oral s-a, 10%	35–37, 39	37, 39				Hippocampus, perirhinal cortex, entorhinal cortex	-↓ number of microglia -↑ microglia dystrophy -↓ BDNF expression
Sánchez-Marín et al. [89]	Rat	M	Intragastric administration, 3 g/kg, 25%	31–55	62				mPFC, amygdala, hippocampus	-↑ mRNA expression of EC signaling -↑ neuroinflammation
Sánchez-Marín et al. [44]	Rat	M	3 g/kg, 20%, i.p.	31–55	62–70	-Anxiety -Working memory	-OF, EPM -NOR	-↑ ALB -Impaired performance	mPFC, amygdala, striatum	-↑ CB1 and CB2 protein expression -↑ CRF receptor protein expression -Region-specific changes in expression of cannabinoid synthetic enzymes
Peñasco et al. [90]	Mouse	M	Oral s-a, 20%	32–56	67	Cognition	NOR	Impaired performance	Hippocampus	-↓ CB1 expression -Impaired CB1 dependent LTD

Note: *PND* post-natal days, *M* male, *F* female, *s-a* self-administration, *i.p* intraperitoneal, *EZM* elevated zero maze, *FST* forced swim test, *EPM* elevated plus maze, *LDB* light dark box, *2 BC* 2-bottle choice, *OF* open field, *NOR* novel object recognition, *ALB* anxiety-like behavior, *DLB* depression-like behavior, *VO-PFC* ventral orbital-PFC, *EPSC* excitatory postsynaptic current, empty spaces indicate information that was not relevant to study or was not specified.

**Table 2:** Results and details of recent animal studies investigating ACE.

Reference	Species	Sex	Route of Administration, Dose	Age of Exposure (PND)	Age at Test(s) (PND)	Behavior Category	Behavior Test(s)	Behavioral Results	Brain Region(s)	Results
Lee et al. [118]	Mouse	M, F	5 mg/kg, i.p.	30–44	70					Alteration in genes associated with microglia homeostasis and immunity
Wan et al. [119]	Mouse	M	2 mg/kg, i.p. (WIN)	33–45	70-71				mPFC, hippocampus, NAc	↑ Iba1 expression in mPFC and NAc following administration of lipopolysaccharide
Renard et al. [125]	Rat	M	2.5, 5, 10 mg/kg, i.p. (2x daily)	35–46	75	-Working memory -Anxiety	-NOR -LDB, OF	-Impaired working memory -↑ ALB	PFC, VTA	-↓ GAD67 expression in mPFC -↑mPFC PN firing rate -↑ VTA dopamine neuron firing
Gabaglio et al. [128]	Rat	F	2.5, 5, 10 mg/kg, i.p. (2x daily)	35–45	75	-Working memory -Depression -Anxiety	-NOR -FST, Sucrose intake -EPM	-Impaired working memory -↑ DLB -↑ALB	PFC	↓ GAD67 expression
Renard et al. [130]	Rat		2.5, 5, 10 mg/kg, i.p. (2x daily)	35–45	75	-Anxiety -Fear	-EPM, OF -Prepulse startle inhibition	-↑ ALB - ↑ prepulse inhibition	VTA	↑ VTA DA neuron firing
Scherma et al. [131]	Rat	M	2.5, 5, 10 mg/kg, i.p. (2x daily)	45–55	70	-Drug taking -Anxiety -Fear -Depression	- WIN s-a -EPM -Prepulse startle inhibition -Sucrose preference test	-↑ s-a -No changes in ALB, DLB, or fear	VTA, NaC shell	-↓ WIN-induced DA activity in VTA -↑ WIN-induced DA activity in NAc shell
Kruse et al. [133]	Rat	M,F	THC gelatin consumption 1.0, 1.5, and 2.0 mg/15 ml	25–58	93	Reward learning	Pavlovian conditioned approach	-↑ Pavlovian conditioned approach in males	VTA	↓ of CB1 expressing VGLUT1 synaptic terminals in males
Friend et al. [263]	Mouse	M	10 mg/kg, i.p.	14.5–23.5	23.5				VTA	Interference of LTD in GABAergic cells
Zamberletti et al. [264]	Rat	M	2.5, 5, 10 mg/kg, i.p. (2x daily)	35–45	75	-Memory -Depression	-NOR -FST	-Impaired performance -No effect	Hippocampus, PFC	-↑ GluN2B, GluA1, and GluA2 expression in hippocampus -↑ astrocytic neuroinflammation in PFC
Le et al. [134]	Rat, Mouse	M, F	5 mg/kg, i.p.	30–43	80	Memory	Serial “what” task, 2-odor discrimination, OLM	Impaired Performance	Hippocampus	↓ potentiation of S-C projection to CA1 in females only
Zuo et al. [136]	Mouse	M, F	10 mg/kg, i.p.	28–48	70	-Anxiety -Memory	-EPM -Six different object test	-No effect -Impaired working memory in females	Amygdala, DMS	Larger number of DEGs in females
Orihuel et al. [138]	Rat	M, F	3 mg/kg, i.p.	28–48 (every other day)	90	-Reward learning	-Pavlovian conditioned approach	-Increased goal-tracking -Increased PIT	NAc shell	Sex-specific changes in transcriptome profile
Chen & Mackie [141]	Mouse	M, F	I.P. injection 10 ml/kg	28–49	65	-Working memory -Anxiety	-Delayed alternation T maze -EPM	-Impaired working memory -No effect		
Withey et al. [142]	Squirrel Monkey	M	Intramuscular Injection, 1 mg/kg	27–31 months	NS	Cognition	Discrimination leaning, discrimination reversal	Impaired performance		
Verrico et al. [143]	Rhesus Monkey	M	Intravenous s-a 15–240 μg/kg	28.6–33.6 months	29 months	Working memory	Delayed match to sample	Impaired performance		
Prini et al. [155]	Rat	F	I.P. Injection 2.5, 5, 10 mg/kg (2x daily)	35–45	45, 46, 47				Hippocampus, NAc, PFC	Transcriptional repression
Prini et al. [156]	Rat	F	2.5, 5, 10 mg/kg, i.p. (2x daily)	35–45	45, 46, 47, 70	Working memory	NOR	Impaired Performance	PFC	-Histone modification -↑ H3K9me3
Miller et al. [157]	Rat	M	1.5 mg/kg, i.p.	28–48 (every third day)	49, 63				PL	↑ dendritic spine pruning, altered gene networks
Leishman et al. [158]	Mouse	F	3 mg/kg, i.p.	35, 50	35, 50				Hippocampus	Changes in transcriptome
Stringfield & Torregrossa [22]	Rat	M, F	3, 10, 30, 100 μg/kg/infusion	32–51	85	Working memory	Delayed match to sample	Impaired performance in females, improved performance in males	PFC, DH, VTA	↓ in CB1, GABA, and glutamate receptor protein
Kirschmann et al. [161]	Rat	F	-WIN intravenous s-a, 0.0125 mg/kg/infusion -WIN, 0.2, 1.2 mg/kg, i.p.	-34–59 -34–53	60, 54	Working memory	NOR, Delayed match to sample	-No effect of s-a -Improved working memory		
Kirschmann et al. [162]	Rat	M	-WIN intravenous s-a, 0.0125 mg/kg/infusion -WIN, 0.2 mg/kg, i.p.	38–49	110	Working memory	NOR, Delayed match to sample	Improved performance	PFC	↑ in expression of proteins regulating GABAergic and glutamatergic signaling
Bruijnzeel et al. [163]	Rat	M, F	-Cannabis smoke exposure, 5.6% THC, 0% CBD -2.5, 5, 10 mg/kg, i.p. (2x daily)	-29–49 -35–45	70	-Anxiety -Depression -Working memory	-OF, EPM -Sucrose preference, FST -NOR	No effect		
Hernandez et al. [164]	Rat	M	Cannabis smoke exposure 5.6% THC, 0% CBD	29–49	70	-Anxiety -Addiction-like behavior	-EPM, OF -Progressive ratio, set shift task	No effect		

Note: *PND* post-natal days, *M* male, *F* female, *s-a* self-administration, *i.p* intraperitoneal, *EZM* elevated zero maze, *FST* forced swim test, *EPM* elevated plus maze, *LDB* light dark box, *2 BC* 2-bottle choice, *OF* open field, *NOR* novel object recognition, *OLM* object location memory, *ALB* anxiety-like behavior, *DLB* depression-like behavior, *PIT* Pavlovian instrumental transfer, empty spaces indicate information that was not relevant to study or was not specified.

**Table 3:** Results and details of recent animal studies investigating ANE.

Reference	Species	Sex	Route of Administration, Dose	Age of Exposure (PND)	Age at Test(s) (PND)	Behavior Category	Behavior Test(s)	Behavioral Results	Brain Region(s)	Results
Corongiu et al. [189]	Rat	M	0.4 mg/kg s.c.	35, 42, 49	25, 42, 49				NAc shell. DLS	-↑ extracellular DA - No effect
Cardenas & Loftipour [181]	Rat	M,F	20 µg/kg/.1 ml, intravenous	28–31	32–36	Drug taking	Meth s-a	↑ meth s-a in males, but not females		
Locker et al. [182]	Mouse	F	Oral s-a, 200 µg/ml	35–41	42–44	Drug taking	Alcohol s-a	↑ alcohol s-a	Frontal cortex	↑ nAChR density
Cardenas et al. [183]	Rat	M,F	30 µg/kg/.1 ml, intravenous	28–31	32	Drug taking	Cocaine and fentanyl s-a	↑ drug s-a		
Reed & Izenwasser al. [184]	Rat	M	0.4 mg.kg i.p.	30–36	39–45	Drug Taking	Cocaine s-a	↑ cocaine s-a		
Lenoir et al. [190]	Rat	M, F	0.001-1 mg/kg i.p.	35–37	35–37, 38	Conditioned reward	CPP	↑ CPP in adolescents than adults	NAc, caudate putamen	↑ nAChR density
Cao et al. [191]	Rat	M, F	30 µg/kg/100 µl intravenous	28, 38	28, 38	Drug reward	Locomotor activity	↑ locomotor activity in adolescents than adults		
Renda et al. [192]	Mouse	M	200 µg/ml Oral s-a	44.4–49.5, 54–59, 60–65	44.4–49.5, 54–59, 60–65	Drug consumption	2 BC	↑ consumption in adolescents than adults	VTA	↑ α4* NACHR in adolescent than adults, correlation between nicotine consume and α4* NACHR expression
Schassburger et al. [194]	Rat	M,F	3, 10, 30 µg/kg/infusion, intravenous s-a	30–46	30–46	Drug consumption	Instrumental responding	↑ responding in adults than in adolescents		
Weaver et al. [195]	Rat	M	0.32 mg/kg s.c.	29–42	64	Reward learning	Instrumental responding for reward cue	No impact of ANE on responding in adulthood		
Jobson et al. [172]	Rat	M	0.4 mg/kg s.c., (3x daily)	35–44	76	-Anxiety -Depression	-OF, LDB, sub-threshold fear memory -FST,	-↑ ALB -↑ DLB	VTA, PFC	-↑ firing of VTA and PFC DA neurons -↓ PFC D1 protein expression -↑ PFC pERK1-2 expression
Hudson et al. [198]	Rat	M	0.4 mg/kg s.c.	35–44 (3x daily)	75	-Anxiety -Depression	-OF, LDB -FST, Sucrose Preference	-↑ ALB -↓ DLB	NAc shell	-↑ pERK1-2 expression -↑ GSK-3 phosphorylation -↓ D1 expression -Altered MSN firing rate
Counotte et al. [205]	Rat	M	0.4 mg/kg s.c.	34–43 (3x daily)	78	Attention	5-choice serial reaction time task	Impaired performance	PFC	↓ mGluR2 protein expression and function
Xue et al. [220]	Rat	M, F	0.03, 0.1, 0.1, mg/kg, s.c.	50–54	50–54	Reward	Intracranial self-stimulation	↓ reward threshold in both sexes, greater reduction in females		
Chellian et al. [221]	Rat	M, F	-NRC 200 (0.07 mg/cigarette)/NRC 600 (0.84 mg/cig) smoke exposure - 0.3 mg/kg, s.c.	24–42	55	Drug taking	Nicotine s-a	↑ s-a nicotine intake in females but not males		
Goriounova & Mansvelder et al. [206]	Rat		0.4 mg/kg s.c.	34–43 (3x daily)	46, 78				mPFC	Altered spike-timed dependent plasticity
Holliday et al. [196]	Mouse	M	0.25 µl/hour s.c. osmotic pump	32–44	45, 74	-Anxiety -Depression -Cognition	-EPM -FST -Contextual fear conditioning	-↑ ALB -↑ DLB - Impaired contextual freezing	CA1	↓ dendritic spine length and complexity
Gitik et al. [209]	Mouse	M	0.25 µl/hour s.c. osmotic pump	23, 38–35, 50	68, 80	Cognition	Contextual fear conditioning	↓ contextual freezing	Hippocampus	Altered methylation of chromatin remodeling genes
Connor & Gould [207]	Mouse	M	12.6 mg/kg/day, s.c. osmotic pump	38, 38–68	70	Learning	Trace fear conditioning	↓ fear learning	Dorsal hippocampus	↓ BDNF expression
Linker et al. [216]	Rat	M, F	30 µg/kg/.1 ml intravenous	28–31 (2x daily)	32	Drug taking	Cocaine s-a	↑ acquisition of cocaine s-a	NAc, BLA	-↑ in IBA+ cells and IBA1 expression

Note: *PND* post-natal days, *M* male, *F* female, *NRC* nicotine research cigarette, *s-a* self-administration, *i.p* intraperitoneal, *s.c.* subcutaneous, *FST* forced swim test, *EPM* elevated plus maze, *LDB* light dark box, *2 BC* 2-bottle choice, *OF* open field, *ALB* anxiety-like behavior, *DLB* depression-like behavior, *DLS* dorsal lateral striatum, *CPP* conditioned place preference, empty spaces indicate information that was not relevant to study or was not specified.

**Table 4:** Results and details of recent animal studies investigating polysubstance use.

Reference	Species	Sex	Route of Administration, Dose	Age of Exposure (PND)	Age at Test(s) (PND)	Behavior Category	Behavioral Test(s)	Behavioral Results	Brain Region	Results
Hamidullah et al. [231]	Rat	M	Alcohol: Oral s-a, 10% THC: Vapor, 10 mg/pad	28–42	28	Drug Consumption	2 BC	↑ preference for alcohol when THC is absent		
Smiley et al. [233]	Rat	M	-THC: Vapor, 200 mg/ml THC + 20 mg/ml CBD - Alcohol: Vapor	-45–49 -50–62	64	Fear	Pavlovian fear conditioning	↑ Freezing, no additive effect of THC and alcohol	PL	↑ calcium activity in response to shock
Lárraga et al. [239]	Rat	M, F	-Alcohol: Intravenous s-a (1–100 mg/kg/infusion) -Nicotine (7.5–30 µg/kg/infusion) solution	32–41	48	Drug Consumption	S-A, 2 BC for alcohol	↑ alcohol consumption in males, no effect in females		
Cross & Leslie [240]	Rat	M	Intravenous injection of nicotine (30 µg/kg) + alcohol (4 mg/kg) solution	32	32	Drug reward, anxiety	OF	↑ locomotor activity, ↓ALB	Posterior VTA	↑ *cFos* expression in adults, no effect in adolescents
Ruffolo et al. [241]	Rat	M, F	Alcohol: oral s-a, 10% Nicotine: Vapor, 5%	30–46	72	-Reward learning -Fear learning	-Sign-tracking -Contextual fear conditioning	-↑ sign-tracking in males -↓ freezing -No additive drug effects		
Dukes et al. [243]	Mouse	M, F	WIN: 0.2, 2 mg/kg i.p. Nicotine: 0.36 mg/kg, s.c.	38–49	70	Drug consumption	Nicotine s-a	↑ nicotine s-a in males, ↑ nicotine s-a in females		

Note: *PND* post-natal days, *M* male, *F* female, *s-a* self-administration, *i.p* intraperitoneal, *s.c.* subcutaneous, *2 BC* 2-bottle choice, *OF* open field, *ALB* anxiety-like behavior, *DLB* depression-like behavior, empty spaces indicate information that was not relevant to study or was not specified.

**Table 5:** Results and details of recent studies investigating human adolescent substance use.

Reference	Substance(s) studied	Age of onset	Mean or median age at testing(s)	Number of users (% male)	Brain Region	Results
Tomek et al. [34]	Alcohol	12–18	18	1,209 (51)		Earlier alcohol initiation predicted later use in females, no effect in males
Squeglia et al. [92]	Alcohol		19.6	75 (60)	Cortex, corpus callosum, pons	-↓ white matter growth - ↑ gray matter reduction
Luciana et al. [93]	Alcohol	16.4	19.2	30	Cortex	-↑ decreases in cortical thickness -↓ white matter development
Meda et al. [94]	Alcohol		20.5	139 (51)	Frontal gyrus, parahippocampus, anterior cingulate	-↓ GMV
Infante et al. [95]	Alcohol		17.5, 18.5, 19.5, 20.5	166 (53)	Frontal regions	-↓ GMV
El Marroun et al. [96]	Alcohol	13.8	16.6	404 (47.8)	Frontal cortex, cingulate cortex	-↓ GMV -↓ increase in WMV
Sun et al. [97]	Alcohol		16.8, 17.7, 18.7	185 (50.8)	Cortex	-Age-dependent changes in cortical thinning
Zhao et al. [98]	Alcohol		20	160 (53.8)	Whole brain	-↓ white matter integrity
Shen et al. [99]	Alcohol		24.8	62 (44)	Whole brain	↓ fractional anisotropy in anterior corpus callosum in males, no effect in females
Phillips et al. [100]	Alcohol	18.8	17.3, 18.3, 19.3	181 (51)	Hippocampus, amygdala	-↓ hippocampal volume -↑ amygdala volume
Murray et al. [111]	Cannabis		19	12 (50)		Adolescents showed greater behavioral and cognitive impairment and decreased P300 amplitude, relative to adults following acute THC exposure
Mustonen et al. [112]	Cannabis	15.5	31.5	375 (44)		↑ risk of psychosis
Penzel et al. [115]	Cannabis	17.4	23.8	102 (67)	Cerebellum	↑ GMV, ↑ positive psychosis symptoms
Subramaniam et al. [127]	Cannabis	15.5	20.4	18 (67)	ACC	↓ GABA levels in adolescent cannabis users
Schaefer et al. [144]	Cannabis	<18	24, 29	3,762		ACE associated with lower educational attainment
Mokrysz et al. [148]	Cannabis		15	526 (47)		ACE not related to IQ or educational performance
Castellanos-Ryan et al. [150]	Cannabis	14–17	20	127 (100)		ACE associated with decline in verbal IQ and executive function tasks, and lower high school graduation.
Albaugh et al. [151]	Cannabis		19	369 (51)	PFC	Accelerated cortical thinning, greater attentional impulsivity
Skumlien et al. [165]	Cannabis		17.1	76 (49)		Lower levels of anhedonia in moderate cannabis users
Skumlien et al. [166]	Cannabis		17.2	32 (50)	Ventral striatum	No change in reward anticipation related activity
Clark et al. [159]	Cannabis		14.3	743 (48)		Identification of methylation markers in problematic cannabis users.
Kinnunen et al. [178]	Nicotine			51 (48)		Nicotine e-cigarette use increases risk of daily cigarette use
Dierker et al. [179]	Nicotine, cannabis	14.7 (cannabis)	18.3	(59)		Nicotine use significantly predicted later cannabis use
Kristjánsson et al. [180]	Nicotine, Alcohol		15.5	587 (49)		Electronic cigarette users were significantly more likely to use alcohol
Treur et al. [210]	Nicotine		15, 42	2,111 (28)		Increase in attentional problems in smokers relative to non-smoking twin
Sylvestre et al. [222]	Nicotine	13	15	839 (44)		Higher likelihood of developing nicotine dependence in girls than boys
Duan et al. [223]	Nicotine		13–18	189 (51)		Higher prvelance of past 30-day smoking in e-cigarette users than non-users. Relationship stronger in boys than girls.
Dai et al. [223]	Nicotine		11.5	116 (52)	Cortex	Impaired cognitive performance, reduced cortical area and volume
Chaarni et al. [214]	Nicotine		14	211	Cortex, corpus callosum	-↓ GMV -Altered corpus callosum connectivity
Yu et al. [215]	Nicotine	15.1	20.8	36 (100)	Thalamus	↓ volume
Luo et al. [234]	Alcohol, Cannabis		16	363 (49)	Whole brain	↓ GMV in alcohol-cannabis co-users than in single-substance users
Infante et al. [235]	Alcohol, Cannabis	16.4 (alcohol), 16.8 (cannabis)	27.5			Impaired cognitive function following AAE and ACE, but no additive effect
Thayer et al. [236]	Alcohol, Cannabis		14–18	201 (74)	Whole brain	↓ GMV in alcohol users, no effect of past 30-day cannabis use
Courtney et al. [244]	Nicotine, Cannabis		16–22	111 (65.5)	Cingulum tract, fornix tract	↓ WMV following nicotine and cannabis poly use

Note. *WMV* white matter volume, empty spaces indicate information not relevant to the study or not specified.

## Alcohol

Alcohol is one of the most commonly used recreational drugs in the world, with adolescents constituting a large group of consumers. However, alcohol has neurotoxic effects and can modify a number of structures and circuits in the brain, including the mesocorticolimbic and striatal systems [26–28]. During adolescence, important changes occur in brain circuits that respond to stress and emotional stimuli, which are sensitive to alcohol exposure [29]. Furthermore, there is a well-established relationship between adolescent alcohol exposure (AAE), brain development, and cognitive functioning [20, 30], as well as data indicating that AAE is associated with increased rate and severity of stress-related psychopathologies [31]. AAE also increases future alcohol consumption in rodents [32, 33], as well as humans [34]. Importantly, adolescents are less sensitive than adults to many of the intoxication cues that suppress drinking, such as motor-impairment, sedation, and hangover, and are more sensitive to the reinforcing effects of alcohol, such as social facilitation [35], which may explain why both human and laboratory animal adolescents will consume more alcohol (relative to body weight) per session of drinking than their adult counterparts [36]. Of equal importance is that repeated AAE can cause neuroinflammation via the release of pro-inflammatory cytokines, which can disrupt synaptic plasticity and lead to neuropathology and cell death [37–39].

In addition, AAE is known to trigger a series of behavioral effects than can often persist into adulthood, many of which are related to anxiety- and depression- like behavior [40–44]. For example, AAE is associated with increased rates of major depressive disorder [45, 46], particularly in females [47]. Studies in animal models suggest that these effects may have multiple sources, one of which is changes in glucocorticoid receptor density and corticotropin-releasing factor (CRF) expression [48]. For example, AAE via two-bottle choice has been shown to increase glucocorticoid receptor densities in the prelimbic cortex (PL), the paraventricular nucleus (PVN), the central amygdala (CeA,) and the basolateral amygdala (BLA) in both late adolescent and adult mice, as well as lead to higher levels of CRF expression in the PVN and CeA in male mice [42]. In addition to directly contributing to the development of anxiety- and depression-like behaviors, AAE can indirectly contribute to the development of anxiety- and depression-like behavior by altering the effect of stress on the brain, particularly in the nucleus accumbens (NAc). Voluntary AAE in rats has been reported to increase dopamine (D)1 receptor expression while decreasing D2 receptor expression in the NAc and alter postsynaptic excitatory signaling following stressors [49]. Epigenetic modifications have also been linked to AAE’s long-lasting effects on anxiety- and depression-like behavior. For example, AAE can lead to long-lasting histone modifications that alter synaptic function in the amygdala and likely contributes anxiety-like behavior [50]. Recent evidence points to AAE-induced epigenetic repression of the synaptic activity response element (SARE) within the immediate-early gene activity-regulated cytoskeleton-associated protein (*Arc*) in the central amygdala (CeA) [51, 52] as a critical mediator for anxiety-like behavior. Bohnsack and colleagues [52] found that restoring histone acetylation at the *Arc* SARE site of the CeA following voluntary AAE in male rats caused a reduction in anxiety-like behavior and excessive drinking to control levels.

AAE also has long-lasting effects on cognitive abilities [53]. Recent studies have reported deficits in recall of an extinguished fear response [54], deficits in reversal learning [43], and impaired working memory [55, 56] following AAE. Unsurprisingly, alcohol’s effect on the medial prefrontal cortex (mPFC) is an important factor in producing these cognitive deficits [57]. Indeed, AAE has been reported to cause a myriad of effects in the mPFC, including greater PL spine density [54], decreased infralimbic cortex (IL) spine density [58], altered PL pyramidal neuron excitability [55, 59], activation of microglia and pro-inflammatory factors [60], decreases in resting state connectivity between PFC subregions [61], and decreases in myelinated fiber density (in males but not females) [62]. AAE also impacts the hippocampus, which is critical in supporting cognitive abilities [63]. Researchers have found that AAE inhibits neurogenesis throughout the hippocampus [64, 65], produces long-lasting reductions in dendritic spine density and alterations in morphology [66], increased levels of astrocytic glial fibrillary acidic protein (GFAP), and decreased levels of brain-derived neurotrophic factor (BDNF) [56].

Potential mechanisms underlying the long-lasting effects of AAE have begun to emerge, one of which is glial functioning and morphology [67, 68]. Astrocyte morphology and astrocyte-neuronal proximity undergo developmental changes, which may make astrocytes vulnerable to AAE [69]. The hippocampus seems to be a critical target for these effects. AAE produces long-lasting alterations to astrocyte activity in the hippocampus [70, 71] and diminishes astrocytic synaptic contact in hippocampal CA1 [72]. Furthermore, AAE elevates levels of astrocytic glutamate transporter (GLT)-1 in the dorsal hippocampus (DH), as well as the ventral hippocampus (VH), in both male and female rats [68]. This finding is particularly relevant given the evidence suggesting AAE alters glutamatergic function [73, 74], and increases in hippocampal glutamate have been linked to schizophrenia [75] and psychosis [76]. However, the effects of AAE are not limited to the hippocampus. Adolescent injections of alcohol in rats also cause changes in PFC subregion astrocyte morphology in the anterior cingulate cortex (ACC) and ventral orbital frontal cortex (vOFC) [77], implying that AAE’s cognitive effects come from changes to multiple brain regions.

Microglia have also been shown to be important mediators of alcohol’s neurotoxic effects [78], in addition to their known role in adolescent brain development [79]. Thus, alcohol’s effects on microglia signaling could lead to a wide array of long-lasting effects. Similar to astrocytes, hippocampal microglia seem to be particularly vulnerable to alcohol. AAE via gavage in adolescent male rats has been shown to produce dramatic increases in microglial activation markers in the entorhinal cortex (EC) and the hippocampus [80]. In another recent example, injections of alcohol in male adolescent mice caused significant loss and dystrophy of microglia in the dentate gyrus (DG) [81] and similar results have been reported in the perirhinal cortex and ECin adult and adolescent male rats [82]. While the long-term consequences of AAE’s effect on microglia are still being determined, it is known that microglia-induced systemic inflammation has many consequences including contributing to long-term neurodegenerative disease [83].

Another consequence of AAE may be long-lasting dysregulation of the endocannabinoid system (ECS). The ECS is widespread throughout the central nervous system and is known to play important roles in many cognitive and behavioral processes [84, 85]. Importantly, the ECS has been implicated in alcohol consumption as well as alcohol addiction [86]. There is some evidence to suggest that cannabinoid (CB)1 receptors may be particularly important in adolescent alcohol consumption. Inhibition of CB1 receptors reduced adolescent alcohol intake in male mice down to adult levels, but did not affect alcohol intake in adults [87]. Moreover, Sánchez-Marín and colleagues have reported several effects of AAE via injections on the ECS. Some of their findings include brain-region dependent changes in mRNA levels of endocannabinoid synthetic enzymes in the PFC and amygdala [44], higher mRNA expression of EC signaling in the mPFC and hippocampus [88], and increases in amygdalar CB1 and CB2 receptor expression (all in male rats) [89]. Furthermore, voluntary AAE in male mice produces long-term deficits in CB1 expression in the hippocampus and interferes with CB1-dependent long-term depression (LTD) [90]. These data suggest that AAE has wide-spread effects on the developing ECS, which could contribute to a wide array of behavioral and cognitive effects.

Research in humans is also producing novel evidence of the harmful effects of AAE. It has been known from some time that AAE disrupts changes in neurodevelopmental trajectories [91]. Specifically, there are accelerated decreases in gray matter and attenuated increases in white matter [92, 93] following AAE. One recent analysis of GMV decline in college students found that, over a two-year period, heavy drinkers had more GMV decline than low drinkers in several brain regions, including the inferior/medial gyrus, parahippocampus, and ACC [94]. Others have recently reported similar results regarding cortical and cingulate GMV [95, 96]. Critically, Sun and colleagues [97] reported that AAE accelerated GMV decline particularly in young adolescents relative to older adolescents. Other recent reports have confirmed that AAE has deleterious effects on white matter microstructural integrity [98, 99]. Furthermore, AAE can alter the overall volume of some brain structures. For example, AAE has recently been linked to smaller whole hippocampal volume and increased volume of the right basal nucleus of the amygdala [100].

Human electroencephalography (EEG) research has also found impairments in overall cognitive functioning following AAE. A recent systematic review reported that AAE increases P3 (an event-related potential related to decision making) amplitude during attention, working memory, and inhibition tasks, suggesting that additional resources were needed to correctly complete these tasks [101]. Together these changes in cortical volume and neural functioning are likely to decrease the overall efficiency of the brain, which could potentially lead to long-lasting cognitive and emotional consequences.

## Cannabis

Cannabis is one of the most popular drugs worldwide and initiation of cannabis use commonly occurs in adolescence. Its availability is rapidly increasing with both medical and recreational legality, and its perception as a potentially addictive and harmful substance is decreasing [102]. Despite this, cannabis still has the potential for misuse, which has been shown to have long-term behavioral and biological consequences [103]. While perceived risk has decreased, evidence for a corresponding increase in adolescent use is mixed, with some studies showing increases in adolescent use [104] and others showing no changes [105]. The primary psychoactive effects of cannabis are thought to be mediated by delta-9-tetrahydrocannabinol (THC), while some nonpsychoactive effects may be mediated by cannabidiol (CBD) and other minor cannabinoids and terpenes [106]. Importantly, the concentration of THC in cannabis products has been increasing over the years, leading to a much more potent product than what has previously been available [107], which could also have greater long-term consequences that are yet to be understood. Furthermore, a recent systematic review found increases in both life-time prevalence and past 12-month use of cannabis vaping among adolescents in the United States and Canada [108], emphasizing a need for continued research in this area.

THC primarily acts on the ECS by binding to CB1 and CB2 receptors. As described above, the ECS undergoes important changes during adolescence that are critical to normative development, such that over-use of cannabis during adolescence might interfere with these changes. Adolescent cannabis exposure (ACE) has also been linked to multiple psychiatric disorders. Of great interest to researchers is its role as a risk factor in schizophrenia and psychosis [109], but it has also been linked to depression, anxiety, and addiction [110]. Furthermore, cannabis use has been shown to have stronger acute behavioral and cognitive effects in late adolescent humans (18–20) than in adults (30–40) [111]. In addition, ACE has been noted to have effects on several brain regions, including the PFC, hippocampus, ventral tegmental area (VTA), and striatum (see [22] for a review).

One of the most concerning potential consequences of ACE is its propensity to precipitate first episode psychosis and the development of schizophrenia [112]. A recent report suggests that the risk of psychosis is elevated in adolescents who have consumed cannabis at least five times [113]. ACE may contribute to the development of schizophrenia by interfering with natural changes in GMV that occur throughout adolescence [114]. One recent investigation found that early cannabis use was associated with greater GMV in the cerebellar schizophrenia-related network as well as more severe positive symptoms of recent onset psychosis (ROP) [115]. Interestingly, some argue that microglia could be important contributors to ACE-induced psychosis [116, 117]. Indeed, ACE is known to activate microglia [118, 119], which are responsible for much of the synaptic pruning that reduces GMV during adolescence [120], potentially leading to unhealthy amounts of synaptic pruning and loss of cortical gray matter [117]. Further research is needed in order establish if microglial effects can explain ACE’s relations with schizophrenia. Importantly factors that moderate the relationship between ACE and psychosis are being identified, and include age of onset, frequency of cannabis use, exposure to childhood trauma, concurrent use of other substances, and genetic factors [121]. On the other hand, recent evidence has also emerged suggesting that genetic risk for schizophrenia may be a risk factor for ACE [122], making causal conclusions about the role of ACE in psychosis difficult to determine. More research is needed to clarify how genetic predisposition and ACE interact to increase risk of schizophrenia and psychosis.

One important target of ACE that may influence risk for schizophrenia is GABAergic signaling in the PFC [123], with ACE generally inhibiting GABAergic activity [124]. Renard and colleagues [125] found that adolescent injections of THC reduced levels of the GABA synthesizing enzyme GAD67 in the mPFC in adult male rats, which was coupled with a hyperactive dopaminergic state. Reductions in PFC GABA levels were also observed in female rats following adolescent injections of THC [126], and adolescent cannabis users have been shown to have significantly reduced GABA levels in the anterior cingulate cortex (ACC) [127]. Interestingly, one recent report has shown that while adolescent injections of pure THC in female rats reduced expression of GAD67 in the PFC, injections of THC-CBD combinations actually increased expression [128], suggesting that the interactions between THC and CBD can have complex effects which need further study.

The mesolimbic pathway is also an important target for the effects of cannabis, and the ongoing reorganization of this pathway in adolescence makes it particularly vulnerable to ACE. Mesolimbic activity is critical for reward learning and is involved in the development of substance use disorders [129]. The VTA in particular may be critically important for changes in reward processing induced by ACE. For example, there is evidence that ACE induces a hyperdopaminergic state in the VTA [130]. Conversely, repeated CB1 receptor activation during adolescence has been found to reduce firing of DA neurons in the VTA and DA release in the NAc shell [131]. CB1 receptors in the VTA are located on both GABAergic and glutamatergic synapses [132], meaning both systems have important roles in modulating dopaminergic activity and reward learning. One recent study reported downregulation of VTA CB1 expression in glutamatergic terminals following ACE via THC-containing gelatin consumption in male, but not female adolescent rats, which was affiliated with an increase in the value of reward predictive cues. These authors argued that loss of CB1 receptors on glutamatergic terminals resulted in greater VTA dopamine firing and increased dopamine release in the NAc [133]. Enhanced VTA dopaminergic activity following ACE is also related to inhibition of GABAergic CB1 expressing neurons. Adolescent injections of THC induce synaptic depression of excitatory synapses onto VTA GABAergic neurons, disinhibiting VTA dopamine neurons in male mice. More work needs to be done to understand to complicated balance between dopamine, glutamate, and GABA in the mesolimbic system, particularly after ACE.

In addition, a number of studies report sex differences in the effects of ACE. For example, there are sex-specific disruptions in long-term potentiation (LTP) of the Schaffer-commisural projection to CA1 and in the lateral perforant pathway (LPP) in adolescent mice and rats following injections of THC, with females showing greater LTP impairment than males [134]. The sex differences in the effects of cannabis may be explained by differences in the metabolism of THC [135, 136], and/or by different ACE-induced changes in gene expression networks in the brain. For example, Zuo and colleagues [137] reported that female mice exhibited a larger number of differentially expressed genes (DEGs) across the amygdala and dorsal medial striatum (DMS) compared to males. Differences in DEGs following adolescent THC exposure have also been shown in the NAc [138]. These studies represent just a part of the growing literature on sex differences in outcomes related to cannabis [139].

ACE also has important implications for cognitive function [140]. Many demonstrations of impaired working memory have been observed in rodents. For example, male and female mice show deficits in working memory in adulthood following ACE via experimenter-administered injections [141]. ACE-induced cognitive impairments have also been observed in nonhuman primates. Injections of THC in adolescent male squirrel monkeys impaired performance in a working memory task [142] and injections of THC in adolescent rhesus monkeys impaired spatial working memory [143]. In humans, studies have reported effects of ACE on academic achievement [144], IQ [145], and performance on specific cognitive tasks [146, 147] (but see also [148]). A systematic review of recent papers investigating the cognitive impacts of ACE found significant effects on outcomes such as executive control, working memory, and academic performance [149]. As is often the case in human studies, baseline differences in cognitive performance are also predictive of early cannabis use [150], meaning that trait level cognitive ability and ACE may interact with each other.

In clinical populations, a 5-year longitudinal study found that cannabis use was negatively associated with thickness in the left and right PFC. Furthermore, thinning in the right PFC was associated with attentional impulsiveness [151, 152]. Moreover, a recent systematic review of the neuroimaging literature on adolescent cannabis users noted preliminary evidence for functional and structural alterations in frontoparietal, frontolimbic, frontostriatal, and cerebellar regions [153]. However, other studies have failed to find effects of adolescent cannabis use on brain volume. A recent meta-analysis of 16 studies that examined global brain volume (GBV) of 12–16 year-olds found no evidence of differences in GBV between cannabis users and non-users [154]. Therefore, it will be important for future research to identify underlying causes for these disparate results, such as frequency of use, total years of use, the potency of the cannabis used, and other confounding factors, such as use of other substances.

The mechanisms by which ACE produces long-term effects are not well established, but recent research suggests that epigenetic factors may play a role. For example, ACE in female rats produced age-dependent alterations in histone modifications in several brain regions, including the PFC, amygdala, and hippocampus [155]. The same group has also reported chromatin modifications in the PFC following ACE in female rats, which was associated with cognitive deficits [156]. ACE in male rats causes a disruption in the developmental trajectory of PL pyramidal neuron transcriptomes along with premature pruning of synaptic spines. Furthermore, the alterations in PFC networks observed in the THC-exposed animals were common to subjects with schizophrenia [157]. There have also been reports of age-dependent alterations in the hippocampal transcriptome following acute administration of THC [158]. Evidence of epigenetic changes have also been shown in humans, as Clark and colleagues reported alterations in several DNA methylation sites following ACE [159].

It should be stated that not all of the literature on ACE has produced evidence of deleterious effects. There is emerging evidence that, at least in rodent studies, that detrimental effects of ACE on working memory could be due to the experimenter-administration of high doses of cannabinoids. Studies from our lab were the first to use adolescent self-administration of cannabinoids (THC or the full agonist WIN55,212-2) to investigate long-term effects on adult working memory performance. We found that adolescent THC or WIN55,212-2 self-administration actually led to improved working memory performance in males, while having no effect or a tendency to decrease performance in females [160–162]. Importantly, self-administration versus experimenter-administration of drugs can often produce very different effects based on the stress of injections and having volitional control over the dose consumed [22]. Further research should be conducted in order to solidify our understanding of the role of dose and administration methods regarding ACE and working memory. There have been conflicting reports of the effects of ACE in other domains as well. There are failures to find any effects of cannabis smoke or THC vapor exposures in male and female rats during adolescence using assays for cognition and anxiety-like behaviors [163, 164]. In humans, moderate adolescent cannabis users have reported less anhedonia than nonusers [165]. Another study found no effect of moderate adolescent cannabis use on ventral striatum activity in a test of reward anticipation [166], again indicating the importance of considering dose in analyses of the effects of all drugs.

## Nicotine

While researchers have been addressing the effects of adolescent nicotine exposure (ANE) for decades, the advent of electronic cigarette products has led to a resurgence of nicotine use among adolescents [167, 168], resulting in the development of a new domain in adolescent nicotine research. In addition to the harmful effects of electronic cigarette products on the body [169, 170], nicotine has a myriad of harmful effects on adolescent development, mood, and behavior. ANE is associated with increased mood and anxiety disorder risk [171–174], in part through alterations in mescorticolimbic dopaminergic transmission [172]. Furthermore, there are known impairments in cognitive abilities [175–177]. ANE also increases the likelihood of continued nicotine use [178], cannabis use [179], and alcohol use in humans [180], as well as other drugs in rodents such as methamphetamine [181], alcohol [182], cocaine [183, 184], and fentanyl [183]. Nicotine also modulates multiple neurotransmitter systems, including dopamine, GABA, glutamate, serotonin, and acetylcholine, all of which require regulation by precise neurodevelopmental mechanisms [185].

Nicotine produces reinforcing effects primarily through its action on the nicotinic acetylcholine receptor (nAchR) system, which is still immature during adolescence [186]. For example, α4β2 and α7 nAchR expression and binding are higher in adolescents than adults [187, 188]. This suggests that adolescents may be particularly vulnerable to the reinforcing properties of nicotine, which are likely mediated through mesocorticolimbic structures. Nicotine injections induce greater DA release in the NAc shell (NAcSh) and NAc core of adolescents relative to adults [189] and adolescents of both sexes show stronger conditioned place preference (CPP) than adults following nicotine injections [190], as well as enhanced locomotor activity following nicotine self-administration [191]. Furthermore, nicotine self-administration has been shown to be greater in male adolescent mice than in adults, which correlated strongly with α4 nAchR expression in the VTA [192]. In humans, adolescent smokers display increased reward-related activity in the NAc, insula, and mPFC [193]. However, studies using self-administration models do not always support the conclusion that nicotine is more reinforcing for adolescents than adults. For example, there is a report of adolescent male and female rats self-administered less nicotine than adults at a low dose [194], and that there are minimal long-term impacts of ANE on reinforcement enhancement [195]. The discrepancy in these results again highlights the importance of considering the method of drug administration and drug dose in interpreting experimental results.

ANE has also been implicated in the development of anxiety- and depression-like symptoms [172, 196–198]. Many of ANE’s effects on anxiety and depression-like behavior are thought to be related to changes in mesolimbic function [185]. One important report comes from Jobson and colleagues [172]. These authors injected male rats with nicotine during adolescence or adulthood and found increases in anxiety-like behavior specifically in adolescent-exposed animals. Furthermore, they reported that ANE resulted in a hyperdopaminergic state during adulthood in the VTA and hyperexcitability of PFC pyramidal neurons (PNs). Importantly, disturbances in mesolimbic DA transmission and hyperexcitability of PFC PNs are both associated with mood and anxiety disorder symptoms [199, 200]. In addition, the authors reported that ANE produced a downregulation in D1 receptor expression and upregulation in phosphorylated extracellular-signal-related kinase (pERK) 1–2 molecular signaling pathways in the PFC, both of which have been associated with increases in anxiety-like behavior [201, 202].

Hudson and colleagues [198] expanded on this by providing evidence for upregulation of NAcSh biomarkers of ANE-induced anxiety-like behavior. The authors gave male rats injections of nicotine during adolescence or adulthood and found several consequences that were only present in adolescent-exposed animals. In addition to increases in anxiety-like behavior, the adolescent-exposed animals displayed several anxiety-related biomarkers, including upregulation of the ERK 1–2 and the protein-kinase B (Akt) glycogen-synthase-kinase-3 (GSK-3) signaling pathways in the NAcSh, as well as a large decrease in D1 receptor expression. Critically, these authors found that selectively targeting the Akt-GSK-3 and ERK 1–2 signaling pathways in the NAc was sufficient to ablate the effects of ANE on anxiety-like behavior, implying that these signaling pathways could be important targets for therapeutic interventions.

ANE has also been shown to produce cognitive deficits, including effects in humans on working memory, attention, and PFC activation [176, 203, 204]. Many of the cognitive effects of ANE are likely mediated by its effects on the mPFC. ANE has been reported to cause short-term increases and long-term decreases in synaptic metabotropic glutamate receptor (mGluR)2 in the rat mPFC, which was associated with deficits in attention [205]. These changes in mGluR2 expression are also thought to mediate changes in pre and postsynaptic activity in the mPFC [206]. Other research points to a role for the hippocampus in ANE-induced cognitive deficits [196, 207, 208]. For example, subcutaneous nicotine delivery in male rats via osmotic pump reduced dendritic length and complexity in CA1 branches of pyramidal cells at adulthood [196]. Moreover, in male mice ANE was shown to alter hippocampal gene methylation and expression in adulthood. Interestingly, administration of the essential nutrient choline ameliorated the ANE-induced decrements in fear conditioning as well as the epigenetic changes in the hippocampus [209], suggesting that choline may have potential as a ANE therapy.

Research in humans generally corroborates the findings from the preclinical literature. ANE is associated with long-lasting effects on attention [210], risky sexual behavior [211], and progression to traditional cigarettes [212]. However, as is the case with much human research, directionality of the effect is subject to debate and more research is needed. In terms of the effects of ANE on the brain, initiation of tobacco use in late childhood has been associated with inferior cognitive performance and smaller cortical volume in frontal, parietal, and temporal lobes at a 2-year follow-up [213]. Moreover, even exposure to low doses of nicotine have been linked to volume reductions in the ventral medial PFC (vmPFC) and altered connectivity in the corpus callosum [214]. There are also reductions in thalamus volume following ANE [215]. In addition, there are alterations in both right dorsal and left ventral frontostriatal tracts in young adult male smokers, which corresponded with poorer performance in cognitive tasks [187, 188]. All of these alterations in brain structure and volume are likely to contribute to the long-lasting effect of ANE on cognition and mood disorders.

In addition, microglial signaling is emerging as an important participant in the long-term effects of ANE, and there are important differences in the effects of ANE on adults versus adolescents. For example, while nicotine inhibits microglia activation in adults, the opposite effect has been found in adolescents [186]. Linker and colleagues [216] reported a myriad of differences in how microglia in adult and adolescent rats respond to injections of nicotine, including differences in IBA1 expression in the NAc and BLA and microglial morphology. Importantly, the authors reported that NAc D2 receptors were responsible for nicotine-induced increases in microglial activation in adolescent rats, as well as the increased cocaine self-administration that was also observed. Furthermore, the effects of ANE on microglia may not be limited to reward-related circuitry. Exposure to nicotine and e-cigarette aerosols during pregnancy have been shown to upregulate hippocampal microglia [217, 218], which is likely to have long-lasting impacts on learning and memory. However, whether these results will hold with ANE is still unknown.

Finally, sex differences surrounding adolescent nicotine have been a subject of interest for some time, with results generally suggesting that females are more susceptible to nicotine’s effects than males [219]. One recent study reported that adolescent nicotine injections reduced brain reward threshold (via intracranial self-stimulation) more in adolescent female rats than males [220]. Another study found that exposure to high-nicotine tobacco smoke increased later nicotine self-administration in adolescent female rats but not males [221]. In humans, adolescent females that smoke are more likely to develop nicotine dependence than males [222]. However, another study reported that male e-cigarette users were more likely than female e-cigarette users to report past 30-day cigarette use at a one-year follow up [223]. More research is needed to make sense of these conflicting reports.

## Poly use

Researchers are increasingly studying the effects of drugs in combination to better model the way humans consume drugs [224–227]. However, this approach is still novel and there is much that we do not know. Alcohol, cannabis, and nicotine use are often directly related to each other [226, 228] with all three often used in combination. Even use of one substance during adolescence can be predictive of use of other substances later in life [229]. Moreover, these drugs can serve to augment the reinforcing properties of each other and continued use of one drug is predictive of relapse of another [230]. When taken in combination, drugs can often have unexpected effects on the brain and body, making it critical to examine the effects of these drugs when they are taken in combination. However, the complexity and novelty of the field has led to some contradictory and unexpected results. Continued work is necessary to gain a full understanding of the effects of polysubstance use.

One recent review of studies investigating individuals that use both cannabis and alcohol reported mixed results, with some findings suggesting that cannabis use may be protective of the effects of alcohol use, while other studies find more negative outcomes such as low academic performance [227]. As human studies in this domain can be difficult to interpret due to factors such as the ratio of alcohol to cannabis use and additional drug use, rodent research has proven highly valuable given the increased experiment control. One study exposed adolescent rats to vaporized cannabis on alternating days while also allowing them to self-administer both alcohol and water in a two-bottle choice paradigm daily. Interestingly, on days when the rats were exposed to cannabis, there was no preference between alcohol and water. However, on days when cannabis was not administered, the rats preferred the alcohol over water [231]. One interpretation is that the rats were increasing their alcohol consumption to compensate for the loss of cannabis, which might replicate substitution effects observed in humans [232]. Other research has examined a possible role for alcohol-cannabis poly use in the development of fear responding. While the combination of THC and alcohol did produce heightened fear responding in male rats as well as greater glutamatergic activity in the PFC, there were not any additive effects compared to cannabis and alcohol alone [233].

There is mixed evidence in humans suggesting additive effects of ACE and AAE. One investigation reported decreases in GMV following poly use of alcohol and cannabis starting in early adolescence, but did not observe this in participants who had exclusively used alcohol or cannabis [234]. However, there is also evidence that the two do not have additive effects in some domains. A 14-year longitudinal study on youth who used both alcohol and cannabis found that there were cognitive performance decrements affiliated with both substances individually, but did not observe additive effects of the two [235]. Furthermore, one recent analysis of GMV and white matter integrity found that alcohol use led to reduced GMV, but that cannabis use in the past 30-days did not have any additional impact on GMV or white matter integrity [236]. Further studies are needed to determine what additive effects AAE and ACE may or may not have.

Examinations of the interactions between alcohol and nicotine are also emerging. Recent analyses of high school e-cigarette users found that a large proportion of their samples reported poly substance use, with alcohol being the most frequently reported drug used in combination with e-cigarettes [237, 238]. There is evidence from rodent studies suggesting that the combination of nicotine and alcohol is more reinforcing in adolescents than adults. One study has found that previous exposure to alcohol and nicotine increased responding for alcohol and alcohol consumption relative to only alcohol exposure, however, this effect was limited to male rats [239]. Furthermore, administration of a low dose of alcohol and nicotine to male adolescent and adult rats produced an increase in locomotor behavior and a decrease in anxiety-like behavior only in the adolescents [240]. Importantly, this experiment did not compare alcohol and nicotine together to the drugs separately, so it is difficult to determine if any additive effects were present. Another study exposed male and female adolescent rats to vaporized nicotine/vehicle vapor and voluntary alcohol/water, which produced long-term changes in reward- and cognition-based behaviors in males but not females, however, they did not find any evidence for additive effects of the drugs [241].

While research examining cannabis-nicotine poly use is limited, recent evidence suggests that e-cigarette use and cannabis use develop in close parallel from middle to late adolescence [242] Studies in rodents have produced interesting sex differences. For example, Dukes and colleagues [243] reported that injections of WIN55,212-2 and nicotine in adolescence increased nicotine self-administration in adult males, but that it decreased nicotine self-administration in females. Courtney and colleagues [244] recently evaluated the effects of cannabis and nicotine poly use on white matter microstructure in a cohort of late adolescents (ages 16–22) and reported that cannabis was associated with white matter reductions, but only when nicotine was also being used. Another study in adults found that the use of both nicotine and cannabis enhanced nAChR availability in the PFC and thalamus to a higher degree than single substance-use alone [245]. More research is needed to corroborate this finding in adolescents, as well as more research on cannabis-nicotine poly use in general.

## Conclusions

Adolescence is a period of both great developmental and behavioral change, making it a particularly vulnerable period for long-term effects of drug exposure. Over consumption of alcohol, cannabis and nicotine, and combinations of these drugs can have long-lasting effects on the brain and body. Continued research is needed to isolate the predictors, mechanisms, and consequences of adolescent drug use, as well as efficacious treatments and pre-use interventions. In particular, many questions still exist around polysubstance use. Further research is needed to establish how these substances pharmacodynamically interact, and how acute and long-term administration of one substance effects consumption of another substance. An understanding of this will enable the use of better models to understand the way that humans consume these substances. Furthermore, we still lack a complete understanding of how adolescent substance use promotes sustained substance use in adulthood. Many theories have been proposed, including dysregulation of the mesolimbic reward system [129], loss of goal-directed control [246], and hyperkaitefia [247], but other mechanisms are likely at work.

Going forward, it will be important to solidify our understanding of the similarities, and the differences of different drug types on adolescents to create therapies that work on the widest possible range of substance use disorders. For example, it has become clear that many drugs can cause proinflammatory central immune signaling, including the acute activation of microglia [248]. This neuroinflammation can have critical long-term effects [67, 249–251] which may contribute to the development of substance-use disorders (see [251] for a review). Targeting neuroinflammation may prove to be an important target for therapies addressing adolescent drug use [252–254]. The EC system also seems to be a common factor related to all three substances. The EC system contributes to the reinforcing effects of all three substances, and undergoes changes following adolescent exposure to them [255]. EC- and inflammation-modulating therapies such as CBD [49, 256, 257] and exercise [258–260] show promise, but more work is needed to determine their efficacy in clinical populations as well as optimal therapeutic protocols. Furthermore, researchers should continue to be aware of potential differences between preclinical studies that use self-administration models of drug taking and studies that use experiment-administered drugs, as the differences in these methods can lead to discrepancies in the dose of drug the animal consumes and produce conflicting results [22, 161]. Furthermore, experimenter-administered methods of drug administration, such as injection or gavage, could be a stressful experience with the ability to induce immune effects in rodents [261], which must be considered in the interpretation of results. Finally, while great progress has been made in including both males and females in research samples, many of the studies mentioned above exclusively studied males (particularly the preclinical studies). Furthermore, an often ignored factor in experimental designs is that male and female rodents (particularly rats) can reach sexual maturity at different ages [262], which can complicate the interpretation of results when male and female adolescent rodents of the same age are compared. Continued emphasis should be placed on the study of both sexes, which has already yielded interesting and important sex differences, and will help to develop more targeted therapeutic interventions.
